# Local anaphor licensing in an SOV language: implications for retrieval strategies

**DOI:** 10.3389/fpsyg.2014.01252

**Published:** 2014-11-05

**Authors:** Dave Kush, Colin Phillips

**Affiliations:** ^1^Haskins LaboratoriesNew Haven, CT, USA; ^2^Linguistics, University of MarylandCollege Park, MD, USA; ^3^Maryland Language Science Center, University of MarylandCollege Park, MD, USA

**Keywords:** memory retrieval, anaphor resolution, Hindi, self-paced reading, computational modeling

## Abstract

Because morphological and syntactic constraints govern the distribution of potential antecedents for local anaphors, local antecedent retrieval might be expected to make equal use of both syntactic and morphological cues. However, previous research (e.g., Dillon et al., 2013) has shown that local antecedent retrieval is not susceptible to the same morphological interference effects observed during the resolution of morphologically-driven grammatical dependencies, such as subject-verb agreement checking (e.g., Pearlmutter et al., 1999). Although this lack of interference has been taken as evidence that syntactic cues are given priority over morphological cues in local antecedent retrieval, the absence of interference could also be the result of a confound in the materials used: the post-verbal position of local anaphors in prior studies may obscure morphological interference that would otherwise be visible if the critical anaphor were in a different position. We investigated the licensing of local anaphors (reciprocals) in Hindi, an SOV language, in order to determine whether pre-verbal anaphors are subject to morphological interference from feature-matching distractors in a way that post-verbal anaphors are not. Computational simulations using a version of the ACT-R parser (Lewis and Vasishth, 2005) predicted that a feature-matching distractor should facilitate the processing of an unlicensed reciprocal if morphological cues are used in antecedent retrieval. In a self-paced reading study we found no evidence that distractors eased processing of an unlicensed reciprocal. However, the presence of a distractor increased difficulty of processing following the reciprocal. We discuss the significance of these results for theories of cue selection in retrieval.

## Introduction

In order to establish grammatical dependencies between words across a distance during routine sentence processing comprehenders rely heavily on their ability to encode and retrieve items from memory. For example, processing of a local anaphor such as the reflexive *themselves* or the reciprocal *each other* in (1) requires recalling the previously seen noun phrase (NP) *the people* from memory so that it may be interpreted as the antecedent.

(1) The people talked to *themselves/each other*.

The mechanism by which previously encountered items are retrieved for subsequent processing has been the subject of recent research. A number of recent studies have motivated a processing model that exploits a cue-based access mechanism to retrieve items from content-addressable memory (e.g., McElree, 2000; McElree et al., 2003; Lewis et al., 2006; Van Dyke, 2007; Martin and McElree, 2008, 2009; Van Dyke and McElree, 2011).

A hallmark property of cue-based retrieval is that it is susceptible to interference (Nairne, 1990). Task-irrelevant items in memory whose features overlap with a probe's retrieval cues (distractors) can exert influence on the retrieval of a target item. In the context of sentence processing retrieval interference is said to occur when grammatically inappropriate distractors influence the processing of a phrase that must enter into a dependency with a previously encountered head. The influence of distractors can be *inhibitory*: a distractor may increase the difficulty of retrieving an appropriate item. Van Dyke (2007) found that distractor NPs increased the difficulty of retrieving a grammatically appropriate subject for the purposes of thematic integration with a verb (see also Van Dyke and McElree, 2006, 2011). A distractor's influence may also be *facilitatory* if its presence decreases the difficulty of processing an otherwise ungrammatical or unlicensed element. Comprehenders have repeatedly showed signs of facilitatory interference during the processing of subject-verb agreement (e.g., Pearlmutter et al., 1999; Wagers et al., 2009). Wagers and colleagues found that reading times immediately following the plural verb *were*, which mismatched the features of the singular subject *key*, were decreased when an intervening distractor [*cabinet(s)*] was plural, compared to when the distractor was singular.

(2) The key to the **cabinet(s)** unsurprisingly *were* rusty from years of disuse.

The authors argued that facilitation arose because comprehenders erroneously retrieved the plural distractor on some portion of trials when attempting to find a licensor for the plural marking on the verb. These kinds of facilitatory interference effects have also been observed in the processing of other grammatical dependencies such as negative polarity item (NPI) licensing (e.g., Drenhaus et al., 2005; Vasishth et al., 2008; Xiang et al., 2009; Parker and Phillips, submitted), and the retrieval of antecedents for null pronominal subjects (PRO) in adjunct clauses (Parker et al., 2012) and many authors have attributed these effects to misretrieval of a distractor under (partial) match with a set of retrieval cues.

Although facilitatory interference has been repeatedly observed in the processing of some dependencies, other dependencies that recruit retrieval have displayed virtual immunity to facilitation from distractors. Recent work has found that the processing of a local anaphor that lacks a grammatical antecedent is unaffected by the morphological feature-content of intervening distractors (e.g., Sturt, 2003; Dillon et al., 2013). For instance, Dillon et al. (2013) demonstrated that the processing of the unlicensed plural reflexive *themselves* in (3) is not influenced by plural-marking on the distractor *manager(s)*.

(3) The new executive who oversaw the manager(s) apparently doubted *themselves*…

The lack of facilitatory interference effects is unexpected on the assumption that the same cues as those used to find licensors for agreement dependencies (e.g., morphological features such as number) are used to identify potential antecedents of reflexives. As with agreement, reflexives must match their licensors in number and gender, so the use of morphological features as cue for retrieval of appropriate antecedents would appear to be motivated. On analogy to agreement licensing, the use of these morphological cues should in turn render antecedent retrieval subject to interference.

The results suggest that morphological features may play a different role in antecedent retrieval for local anaphors than they do in agreement licensing. One option, advocated by Dillon et al. (2013), is that antecedent retrieval forgoes the use of interference-prone morphological features, opting instead to exclusively use *positional* syntactic features to access the local subject. Another option is that antecedent retrieval preferentially weights syntactic cues over morphological cues instead of avoiding them altogether. This second account predicts a small but non-negligible interference effect that the first does not, but previous experiments may not have had sufficient power to find this effect, so they cannot distinguish between the two competing explanations.

Although the two accounts differ, they both assign priority to positional cues. This goes against the general assumption that retrieval identifies targets through the use of a maximal cue set that uniformly weights lexical, morphological, syntactic, and semantic features (see Van Dyke and McElree, 2011 for discussion).

As it stands the previous studies may not be sufficient to establish a preference for positional features. It is possible that the absence of facilitatory interference could be attributed to a confound that masks the contribution of morphological features that are weighted equally to syntactic cues. In almost all previous studies the critical anaphor immediately followed its verb, which could potentially play a role in reducing the incidence of facilitatory interference (see King et al., 2012 for a similar suggestion).

As Dillon et al. (2013) note, the post-verbal position can provide an anaphor with privileged access to the local subject by means of recent activation alone. If subjects are retrieved by their verbs for thematic integration, the local subject *the executive* in (3) should be recalled by the verb *doubted*. Retrieval of the local subject entails that it should have the highest baseline activation out of all other items in memory immediately following the verb. At the time that a verb-adjacent reflexive is encountered, this high degree of activation may be strong enough to guarantee retrieval of the local subject instead of the feature-matching distractor even if morphological cues were used.

Alternatively, it may be that previous studies on reflexives do not provide a measure of susceptibility to facilitatory interference because establishing a dependency between the local subject and a post-verbal anaphor might not require retrieval at all. Some theories assume that the most recently retrieved item is maintained in a state that the parser can access without retrieval. In some theories this state is referred to as the *focus of attention* (e.g., McElree, 2000), in others such as Lewis and Vasishth's (2005) parsing model it is the *problem buffer*. When an anaphor is encountered immediately following the verb, it is possible that it consults the contents of this buffer to find its antecedent rather than initiating a retrieval from memory.

In this study we address the extent to which the lack of facilitatory interference in anaphoric licensing depends on an anaphor's post-verbal position. If the absence of interference is a consequence of the target anaphor occupying an immediately post-verbal position, then in languages where anaphors uniformly precede their verbs, local anaphor licensing should display facilitatory effects that have not been seen in English. We tested this prediction by investigating the processing of Hindi reciprocals. Hindi is a language in which all arguments and adjuncts precede the verb in unmarked word order. In (4), for example, the subject *LaRkoN* (“boys”), the reciprocal object *ek-dusre* (“each other”), and the adjunct *kal* (“tomorrow”) precede the verb *dekhaa* (“saw”).

(4) LaRkoN-ne ek-dusre-ko kal dekhaa.
Boys-Erg each.other-Acc yesterday saw.
‘(The) boys saw each other yesterday.’

Hindi reciprocals provide a minimal contrast to English reflexives because they are subject to nearly identical licensing conditions as English local anaphors. Their antecedent must have matching morphological features: in order to license the reciprocal in (5), the local subject must bear plural features. The reciprocal's antecedent must be contained in the same local clause as the reciprocal: the main clause subject in (6) cannot antecede the reciprocal in the embedded clause, despite bearing correct number marking, because it is not local to the reciprocal. Finally, the reciprocal's antecedent must also c-command the reciprocal (cf. Dayal, 1994). In (7), the plural NP *boys* does not c-command the reciprocal because it is embedded inside the adjunct phrase *at the boys' party*. It is therefore ineligible to license the anaphor.

(5) *LaRk*-{^*^-e/oN}-ne *ek-dusre*-ko kal dekhaa.
Boy-{Sing./Pl.}-Erg each.other-Acc yesterday saw.
‘(The) boy*(s) saw each other yesterday.’
(6) ^*^LaRkoN-ne kahaa ki Mary-ne *ek-dusre*-ko dekhaa.
Boys-Erg said that Mary-Erg each.other-Acc saw.
^*^‘(The) boys said that Mary saw each other.’
(7) ^*^Mary-ne [*larkoN*-ki parTi me] *ek-dusre*-ko dekhaa.
Mary-Erg boys' party in one-another-Acc saw
^*^‘Mary saw each other at the boys' party.’

We test whether morphological number features engender facilitatory interference effects during the processing of Hindi reciprocals.

## Simulations

We ran a series of computational simulations that modeled local anaphor resolution in Hindi using equally-weighted morphological and positional features as cues for retrieval. Modeling was carried out to obtain qualitative predictions about the character and direction of interference from the distractor's morphological features that could then be compared with empirical reading times in the self-paced reading experiment.

### Procedure

We implemented a modified version of Lewis and Vasishth's (2005) ACT-R model of sentence processing [using code originally developed by Badecker and Lewis (2007)]. ACT-R is a general cognitive architecture that has been used to model a wide range of phenomena in cognitive psychology (Anderson, 1990). In the model, items are stored as “chunks” in a content-addressable memory and are retrieved with a success proportional to their overall activation at the time of retrieval, which is in turn determined by the overlap of their features with those of a retrieval probe. Memory access is modeled as a rational procedure that employs a general retrieval mechanism that minimizes retrieval error in the limit (Anderson, 1989; Anderson and Milson, 1989; Anderson and Schooler, 1991). Although fully implemented ACT-R parsing models exist (e.g., Lewis and Vasishth's, 2005 ACT-R parser), the simulations here focus solely on modeling retrieval latencies, abstracting away from the contributions of other modules. Retrieval latencies do not exhaust the processes that must be carried out in order to advance to the next word in a parsing task (other operations include structural attachment and integration), but for current purposes we adopt the standard assumption that longer retrieval latencies entail longer RTs (Anderson and Milson, 1989).

In the model the probability of retrieving an item i is governed by its activation A_i_, computed as in (8). B_i_ is chunk i's baseline activation. The weight assigned to the individual cue j is represented w_j_. For the purposes of our simulations cues were assigned uniform weights, so this term can be effectively dropped. S_ji_ is the strength of association between cue j and chunk i. PM in the equation below is a term that penalizes partial matches. The term ε introduces stochastic noise.

(8) A_i_ = B_i_ Σ w_j_S_ji_ + PM + ε_i_

S_ji_ is calculated according to the Equation in (9), where S is a parameter that specifies the maximum strength of association allowed. The fan_j_ term reflects the number of items that bear cue j. The term provides a way of quantifying the distinctiveness of a particular cue. The fan serves to decrease the associative strength between item i and cue j as a function of the number of total cues in memory that bear j.

(9) S_ji_ = S − ln(fan_j_)

Baseline activation is calculated according to (10), where d is the decay rate of a chunk's activation in memory at a given point since retrieval time t_m_.

(10) B_i_ = ln[Σ_m_ t^−d^_m_]

The chunk with the highest activation has the shortest retrieval latency (T_i_) as calculated according to the equation below, where *F* is a scaling parameter. The chunk with the shortest retrieval latency is the chunk that is retrieved in simulations.

(11) T_i_ = *F*e^−A_i_^

The model equations above contain a number of free parameters whose settings could impact the results of the simulation. We ran a series of simulations that systematically combined parameter values from across the range of those reported in previous work. Values of the *total source activation*, *activation noise*, *fan*, *decay rate*, and *match-penalty* parameters were manipulated^1^. The scaling factor (F) was held constant at 0.75 across all simulations. This resulted in the construction of 324 different models with unique parameter value combinations. As noted by Dillon et al. (2013), conducting such a sweep through the space of possible parameter values and combinations enables the identification of model predictions that are independent of idiosyncratic parameter combinations. 10,000 Monte Carlo simulations were run for each model, providing for each simulation a prediction of the most probable retrieval target and its retrieval latency.

### Materials

We simulated antecedent retrieval time-locked to a position corresponding to the critical reciprocal in a sentence that contained three preceding NPs. The first NP, the *subject*, corresponded to a structurally appropriate antecedent for the reciprocal. The second NP, introduced at a lag after the subject NP, corresponded to a structurally inappropriate distractor. A third NP (NP3) was also introduced to more directly model the materials in our self-paced reading (SPR) experiment, the design of which is discussed below. The three NPs were introduced at 300 ms, 900 ms, and 1500 ms after simulation onset. Retrieval of the critical reciprocal was scheduled at 2400 ms after simulation onset.

Each NP in the simulation was marked with three features relevant for retrieval: its *category*, *number*, and *clause index*. All NPs bore the NP category feature. Number features could be either *singular* or *plural*. The *clause index* feature was used as a proxy feature for encoding an NP's structural appropriateness for the purposes of binding the reciprocal: the local licensing requirement is assumed to be satisfied if the antecedent bears the same clause index as the reciprocal. This indexing scheme can be viewed as a feature-based implementation of the clause-mate constraint on local anaphor licensing (see Lasnik, 2002 for a review of such constraints, which can differ in formulation from the c-command constraints of Chomsky, 1981; Reinhart, 1983).

Models were run to simulate four distinct conditions, corresponding to different feature combinations on the subject and distractor. The number features on the subject and the distractor were manipulated, resulting in the 2 × 2 factorial design schematized in (12). In grammatical conditions the subject was plural-marked, in ungrammatical conditions the subject was singular. In *NoInterference* conditions the distractor was singular, while in *Interference* conditions it was plural-marked. The structurally appropriate subject NP was marked with the *main clause* feature, while both the distractor and NP3 were marked as *embedded* and were therefore ineligible to antecede the reciprocal.

(12)
  - Grammatical-NoInterference
    [Subject]+PL… [Distractor]+SG… [NP3]+SG… [RECIPROCAL]+PL
  - Grammatical-Interference
    [Subject]+PL… [Distractor]+PL… [NP3]+SG… [RECIPROCAL]+PL
  - Ungrammatical-NoInterference
    [Subject]+SG… [Distractor]+SG… [NP3]+SG… [RECIPROCAL]+PL
  - Ungrammatical-Interference
    [Subject]+SG… [Distractor]+PL… [NP3]+SG… [RECIPROCAL]+PL

Antecedent retrieval at the reciprocal was modeled as specifying *NP* as a category cue and *main clause* as the clause cue. The number feature *plural* was also used in the retrieval cue set, to measure the interference effect associated with morphological features.

### Results

We report three measures of interest from the simulations run for each condition: (i) predicted error rate, (ii) average predicted latency by condition, and (iii) predicted interference effect.

Predicted error rate corresponds to the percentage of the runs when the distractor, rather than the appropriate subject, was retrieved as an antecedent for the reciprocal. This measure is a relevant index of facilitatory interference in the ungrammatical conditions if facilitation stems from erroneous retrieval of the distractor instead of an appropriate target NP.

Predicted latency provides a measure of how long on average the winning retrieval should take in each condition. In simulations, the chunk with the shortest retrieval latency is the chunk that is retrieved from memory. According to the fully implemented ACT-R model, reading times on a particular word or phrase are the sum of the latency of retrieval triggered at that phrase and the amount of time associated with subsequent processing required by that word or phrase. Retrieval latencies should therefore map monotonically to reading times, with longer retrieval latencies corresponding to longer overall reading times, although the mental processes that intervene between retrieval and button-press may interact or contribute additional difficulty in such a way as to distort the underlying pattern of retrieval. Despite the possibility of later processing concealing underlying retrieval patterns, previous work has found a degree of relative transparency between the qualitative pattern of retrieval latencies furnished by the model and observed effects of facilitatory interference in self-paced reading or eye-tracking measures (see e.g., Wagers et al., 2009; Dillon et al., 2013).

The interference effect is a difference measure that compares average retrieval latencies between two conditions that differ on a single feature, as a way of estimating the magnitude and direction of interference contributed by the retrieval probe matching that one feature. We report two interference effects: the difference between the two grammatical conditions, as well as the difference between the two ungrammatical conditions. These comparisons provide a quantitative prediction of the effect of distractor plural marking when the features of the appropriate subject are held constant.

### Predicted error rates

Error rates are reported in Table 1. The error rates are consistent with a profile of facilitatory interference. Between the *Ungrammatical* conditions, plural marking on the distractor is predicted to increase rates of erroneous retrieval compared to when there is no NP in the sentence that matches the reciprocal in features (26.1 vs. 6.5%). On some proportion of trials, the recency of the distractor is predicted to increase the NP's baseline level of activation enough to result in it being the most highly-activated NP at retrieval. In the *Ungrammatical-NoInterference* condition, the distractor does not share any features with the reciprocal's cue set, so the main subject is still more likely to be retrieved, as it matches the retrieval probe's clause index cue. Error rate is expected to differ slightly between the two grammatical conditions: misretrieval of the distractor is 5.4% more common when it bears plural marking and the main subject matches the retrieval cues completely.

**Table 1:** **Retrieval error rates by condition for retrieval using morphological and syntactic cues calculated as the percentage of trials on which the distractor was retrieved across 10,000 runs each of 324 different models with unique parameter combinations**.

	**NoInterference (%)**	**Interference (%)**
Grammatical	2.0	7.4
Ungrammatical	6.5	26.1

### Average predicted retrieval latencies

**Figure 1:**
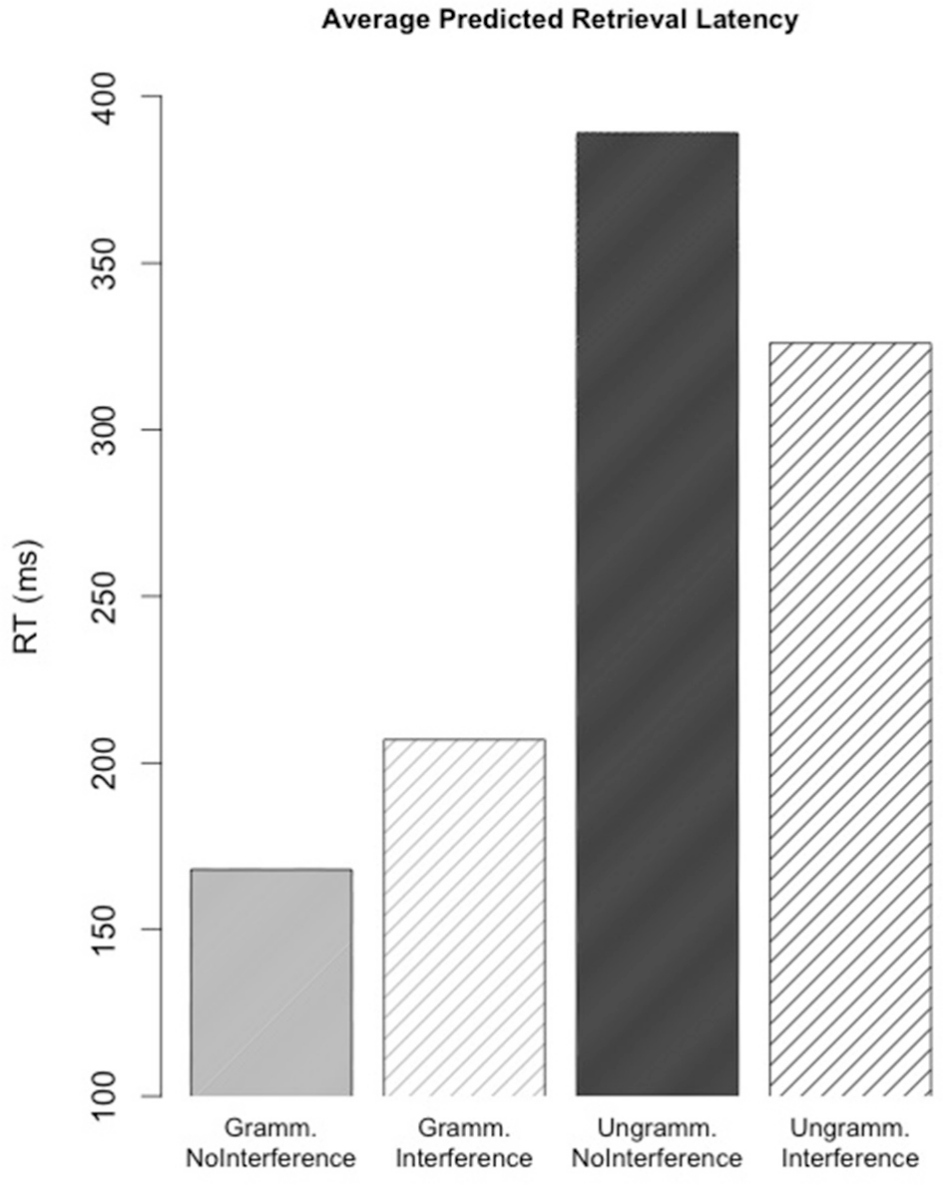
**Retrieval latencies by condition as predicted by the model in Experiment 1**. Reported retrieval latencies represent the mean latency by condition across all simulations.

In the simulations the presence of a feature-matching subject has a facilitative effect on retrieval latencies (see Figure 1). Overall, retrieval times should be faster in the grammatical conditions because the grammatical subject, which matches the reciprocal's morphological and syntactic retrieval cues completely, is highly activated. Increased activation due to greater feature-match with the probe results in faster retrieval latencies in accordance with Equation (11). In the *Ungrammatical* conditions, where the main subject matches only on syntactic cues, retrieval latencies should be longer because the retrieved chunk should never match the probe completely. The appropriate subject only matches the probe's category and positional cues. The distractor matches the category cue and, in the *Ungrammatical-Interference* condition, the reciprocal's number feature. A pairwise difference is also predicted between the average retrieval latencies in the *Ungrammatical-NoInterference* and *Ungrammatical-Interference* conditions, which can be linked to the presence of morphological plural marking on the distractor. On the proportion of trials where the distractor is retrieved in the *Ungrammatical-Interference* condition, latencies are reduced relative to when the main clause subject is retrieved. This results in a reduction of average latency across retrievals.

### Interference effects

Predicted interference effects are shown in Table 2. The grammatical interference effect was calculated by subtracting the average predicted latency in the *Grammatical-Interference* condition from the predicted latency in the *Grammatical-NoInterference* condition. The same difference was calculated for the two ungrammatical conditions. 95% confidence intervals represent the range of predicted interference effects across simulations.

**Table 2:** **Average interference effects across 10,000 runs each of 324 different models**.

	**Interference effect**	**Middle 95% of simulated distributions**
Grammatical	+36 ms	+11, +82 ms
Ungrammatical	−63 ms	−18, −142 ms

The simulation results predict that a plural-marked distractor should cause facilitatory interference in the ungrammatical conditions. The *Ungrammatical-Interference* condition exhibits faster average retrieval latencies than the *Ungrammatical-NoInterference* condition. Though the size of the effect varies, a facilitatory effect was consistently observed across all parameter combinations.

A small effect of inhibitory interference is also predicted in the grammatical conditions. This inhibition can be attributed to the fan effect (see Equation 8). In the *Grammatical-Interference* condition, the strength of association between the appropriate subject and the plural retrieval cue is decreased relative to the *Grammatical-NoInterference* condition, due to the presence of another plural-marked NP (the distractor).

### Discussion

The goal of the simulations was to obtain predictions about the effect that a feature-matching but syntactically inappropriate distractor would have on the retrieval of an antecedent for a local reflexive if that retrieval used morphological features as cues that were weighted equally to syntactic cues.

The simulations show that when morphological cues are assigned the same weight as syntactic cues, the presence of a feature-matching distractor should decrease the parser's ability to retrieve a syntactically appropriate but feature-mismatching subject as an antecedent for a local anaphor. Some proportion of the time, the distractor is expected to be erroneously retrieved as a result of partial overlap with the retrieval cues. This misretrieval is predicted to have a facilitating effect on reading times in comparison to a case of retrieval when neither the distractor nor the local subject match the reflexive.

## Self-paced reading experiment

The modeling results predict that retrieval of a pre-verbal reciprocal's antecedent should display facilitatory interference effects from structurally inappropriate distractors, if morphological cues such as number are assigned the same weight as syntactic cues in retrieval. The experiment below used the self-paced reading method to investigate whether evidence of the predicted facilitatory interference would be found.

### Materials

The experiment had a 2 × 2 factorial design that matched the simulated conditions. The design manipulated the factors Grammaticality and Interference. The structure of the test items is schematized in (13) and an example item is given in (14). All conditions contained a critical reciprocal (*ek-dusre*) that required a plural-marked antecedent in the main clause. The reciprocal was contained in a post-positional phrase that preceded a manner adverbial (*gupt-rup-se*, “*secretly”*) and the main clause verb (*baat kii*, “*chatted” lit.* “*chat did”*).

Grammaticality was manipulated by changing whether the main clause subject was plural-marked [*Doctor(-oN)*, “*doctor(s)”*]. Plural marking was unambiguously marked by the inflectional suffix *–oN*. In the grammatical conditions, the main clause subject was plural and could therefore act as a grammatical antecedent for the reciprocal. In the ungrammatical conditions, the local subject was singular and the reciprocal therefore lacked a clause-mate antecedent. The factor Interference manipulated whether the distractor [*mariiz-(oN)*, “*patient(s)”*] was plural-marked.

In previous studies on local anaphor licensing (e.g., Sturt, 2003; Dillon et al., 2013) distractors have been positioned within relative clauses (RCs) attached to the main clause subject. RC-modification of subjects is a marked construction in Hindi, so the present study embedded the distractor inside a locative phrase that preceded the critical reciprocal.

The locative phrase contained an NP denoting a location modified by an animate possessor (*nurse-ke steSan*, “the nurse's station”). The distractor was embedded as the object of a verb within a prenominal RC that was attached to this possessor. In this position the distractor was not a clause-mate of the reciprocal and was therefore ineligible to act as a potential antecedent.

Critical reciprocals were always followed by a case marking post-position, either the genitive *ke*, the objective *ko*, or the dative *se*. When followed by the genitive, reciprocals were embedded in a complex post-position that was an argument to the main verb (e.g., *ke bare-me* “about” in 14). In sentences with *ko* or *se*, adverbial material was introduced after the post-position to maintain consistent length across sentences.

(13) Subject-{sg/pl} [_PP_[_RC_ Distractor-{sg/pl} V] NP's Location] Reciprocal P Adv V
(14)
  - Grammatical-NoInterference
    DoctoroN-ne mariiz-ki dekhbaal karne-wali nars-ke sTeSan-me ek-dusre ke-bare-me gupt-rup-se baat kii.
    Doctors-Erg patient-Gen care doing-RP nurse's station-in each-other aboutsecretly chat did.
    ‘The doctors secretly spoke about each other in the station of the nurse taking care of (a/the) patient.’
  - Grammatical-Interference
    DoctoroN-ne mariizoN-ki dekhbaal karne-wali nars-ke sTeSan-me ek-dusre ke-bare-me gupt-rup-se baat kii.
    Doctors-Erg patients-Gen care doing-RP nurse's station-in each-other about secretly chat did.
    ‘The doctors secretly spoke about each other in the station of the nurse taking care of (the) patients.’
  - Ungrammatical-NoInterference
    Doctor-ne mariiz-Gen dekhbaal karne-wali nars-ke sTeSan-me ek-dusre ke-bare-me gupt-rup-se baat kii.
    Doctor-Erg patient-ki care doing-RP nurse's station-in each-other about secretly chat did.
    ‘The doctor secretly spoke about each other in the station of the nurse taking care of (a/the) patient.’
  - Ungrammatical-Interference
    Doctor-ne mariizoN-ki dekhbaal karne-wali nars-ke sTeSan-me ek-dusre ke-bare-me gupt-rup-se baat kii.
    Doctor-Erg patients-Gen care doing-RP nurse's station-in each-other about secretly chat did.
    ‘The doctor secretly spoke about each other in the station of the nurse taking care of (the) patients.’

Inside the pre-nominal RC the distractor bore either accusative or genitive case (according to the verb's requirements). Although this increased the contrast between the nominative grammatical subject and the distractor, it is unlikely that the case difference would play a role in distinguishing appropriate from inappropriate NPs, as accusative and genitive-marked NPs can serve as antecedents for local anaphors under the right structural conditions (see, e.g., Dayal, 1994; Mohanan, 1994; Bhatt and Dayal, 2007).

A second concern with the experimental materials is that there exists the potential for temporary misanalysis of the structural position of the distractor during incremental parsing. When it initially encounters the distractor, the parser has not yet encountered any information that indicates that the distractor is contained within an embedded clause. In the absence of this information, an incremental parser is likely to analyze the distractor as a constituent of the main clause. This type of temporary misparse is common in head-final languages where embedded arguments can be encountered prior to the verb that licenses them (Inoue, 1991; Mazuka and Itoh, 1995; Miyamoto, 2003). The misanalysis would be disconfirmed at the relative pronoun *wali*, at which point the object would be correctly reanalyzed as a constituent of the relative clause. This misparse should occur across all conditions, but it may have a greater impact on processing in the *Ungrammatical-Interference* condition. Under this misanalysis the RC-internal object would initially be analyzed as a suitable antecedent for an upcoming reciprocal. We return to the ability of such a misparse to affect later parsing decisions in the *Ungrammatical-Interference* condition in the discussion.

### Participants

32 self-reported native speakers of Hindi were recruited from the student bodies of IIT, Delhi and Jawaharlal Nehru University in New Delhi (18 male, mean age = 20.1). Participants were compensated Rs. 300 for their participation, which lasted around 35 min.

### Procedure

Participants were run on one of two laptop PCs using the Linger software package (Doug Rohde, MIT) in a self-paced word-by-word moving window paradigm (Just et al., 1982). Each trial began with a sentence masked by dashes appearing on the screen. Letters and punctuation marks were masked, but spaces were left unmasked so that word-boundaries were visible. As the participant pressed the spacebar, a new word appeared and the previous word was re-masked. All text appeared in Devanagari font.

A yes/no comprehension question that probed its interpretation followed each sentence (experimental materials can be found at the first author's website). Participants were instructed to read sentences at a natural pace and to respond to the comprehension questions as accurately as possible. Participants responded to questions using the f-key for “yes” and the j-key for “no.” If the question was answered incorrectly the word *galat* (“incorrect/wrong”) appeared briefly in the center of the screen. Each participant was randomly assigned to one of the lists and the order of the stimuli within the presentation list was randomized for each participant.

### Analysis

Data from one participant were excluded due to failure to comply with experimental guidelines. Data from another participant were excluded because the participant's mean accuracy on comprehension questions was close to chance. This resulted in the data of 30 subjects being used for later analysis. Two items were excluded from analysis due to errors.

Statistical analyses were carried out on log-transformed reading times using linear mixed effects regression (Baayen et al., 2008). Reading times from both correct and incorrect trials were included in the analysis. Experimental fixed effects were the simple difference sum-coded factors Grammaticality and Interference and their interaction. All models included random intercepts for both subjects and items. Models with a maximal random effects structure were fit whenever possible (Barr et al., 2013). If a maximal model failed to converge, a model was used that contained only by-subject random slopes for both fixed effects and their interaction.

### Results

#### Comprehension Question Accuracy

Comprehension question accuracy averaged 69.2%. No significant differences were found in average accuracy across conditions (logistic mixed effects model, all *z*s < 1).

#### Reading Time Results

**Figure 2:**
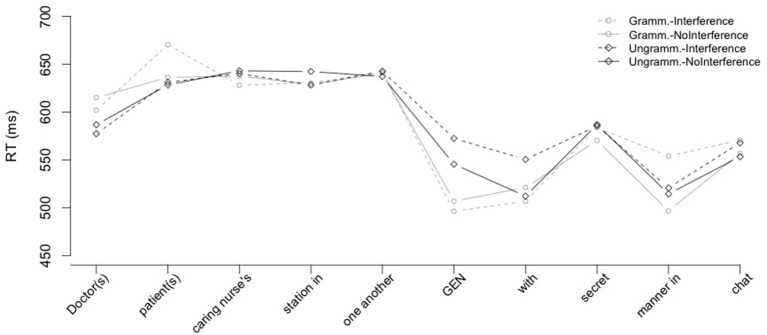
**Average word-by-word self-paced reading times for all items in Experiment 1**.

Reading times from the post-reciprocal region are given in Figure 2.

***Pre-reciprocal region.*** No significant effects were found in the pre-reciprocal region.

***Reciprocal region.*** No significant effects were found in the reciprocal region.

**Figure 3:**
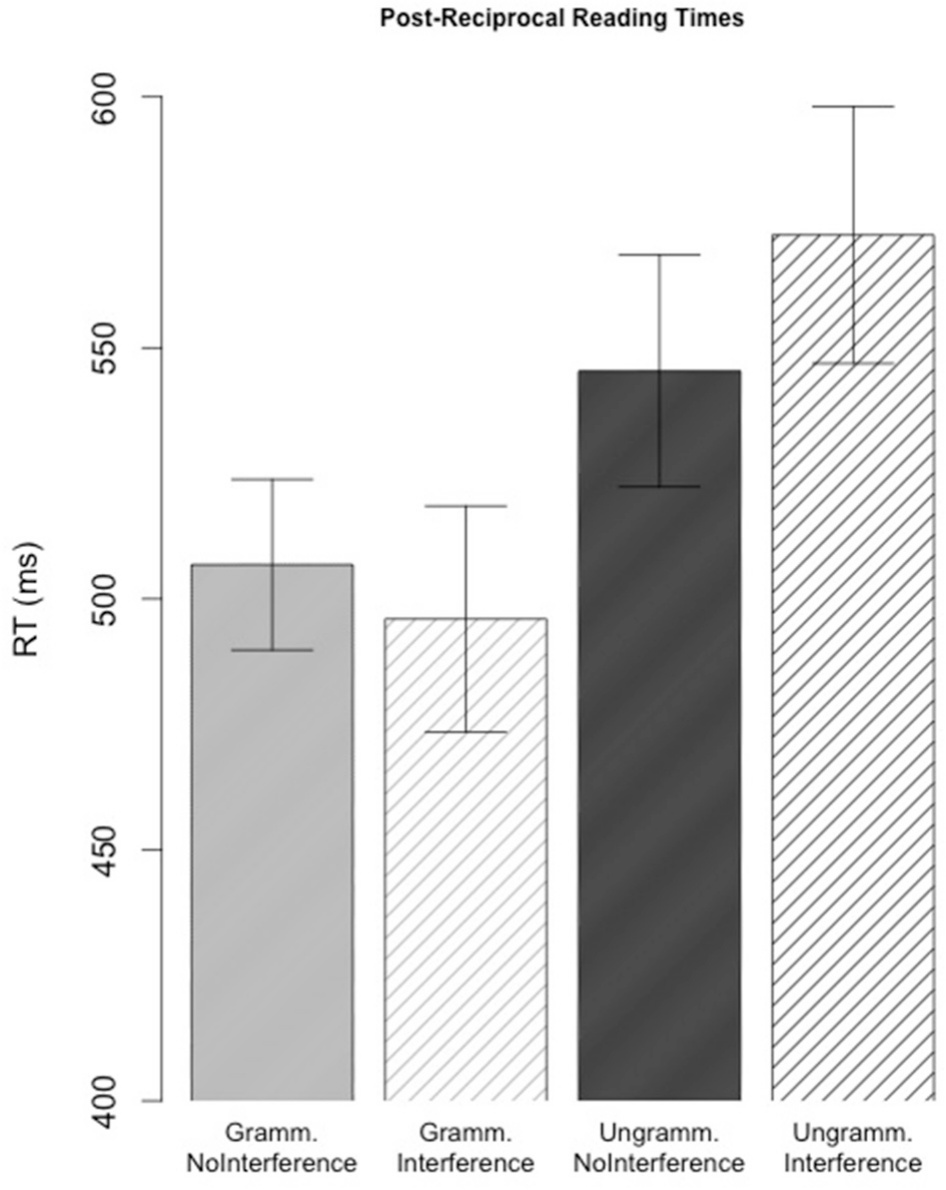
**Average post-reciprocal self-paced reading times in Experiment 1**. Error bars indicate standard error of the participant mean.

***Post-position region.*** Average reading times were reliably faster in the grammatical conditions than in the ungrammatical conditions (main effect of Grammaticality: $\hat{\beta }$ = −0.088, s.e. = 0.034, *t* = −2.92); see Figure 3. Although reading times in the *Ungrammatical-Interference* condition were numerically longer than those in the *Ungrammatical-NoInterference* condition, the Grammaticality × Interference interaction was not significant (*t* = 1.41). No reliable pairwise differences were observed between ungrammatical conditions (*t* < 1).

***Reciprocal+2 region.*** There were no significant main effects two regions after the critical reciprocal, but the model revealed a marginally significant Grammaticality × Interference interaction ($\hat{\beta }$ = 0.105, s.e. = 0.054, *t* = 1.96) two regions after the reciprocal. This interaction reflected the fact that the *Ungrammatical-Interference* condition was read more slowly than any other condition, including the *Ungrammatical-NoInterference* condition. The pairwise comparison between the two ungrammatical conditions revealed the numerical difference between the two conditions not to be significant (*t* = 1.3). However, given the relatively low power of the current study, it is possible that this interaction would achieve significance with higher power. We return to this interaction effect in the discussion.

***Reciprocal+3 till Final region.*** No significant effects were observed in any subsequent region.

### Discussion

The SPR experiment sought to determine whether the processing of a pre-verbal reciprocal in Hindi was subject to facilitatory interference. The study manipulated the number features on a structurally appropriate antecedent for the reciprocal, as well as the features of the structurally inappropriate distractor, as a means of testing whether (equally weighted) morphological cues are used to access a local anaphor's antecedent.

**Figure 4:**
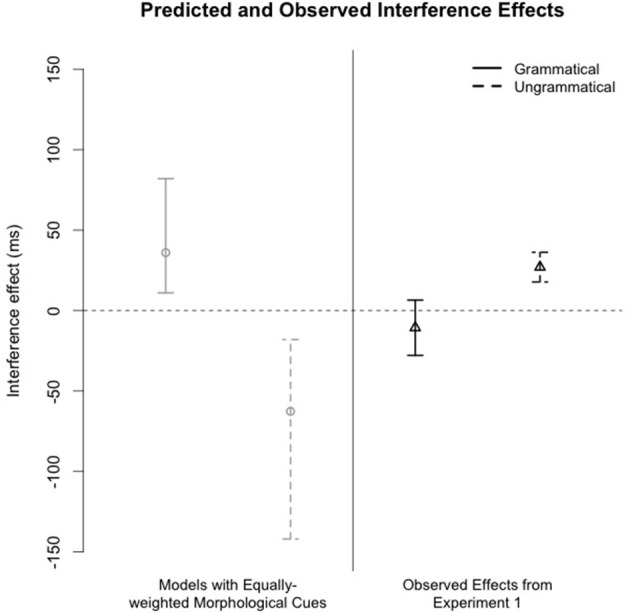
**Comparison of predicted interference effects from model simulations and observed interference effects from Experiment 1**. Models simulated expected retrieval latencies if morphological and positional cues were assigned equal weights in antecedent retrieval. For the simulated data, error bars represent the middle 95% of the distribution of predicted interference effects. Error bars around the empirical means mark the 95% CI.

When a structurally appropriate feature-matching antecedent was present to license the pre-verbal reciprocal the regions following the critical reciprocal were read more rapidly than when there was no feature-matching and structurally appropriate antecedent. In contrast to the prediction of the model simulations, we failed to find any evidence of facilitatory interference (see Figure 4). In fact, the empirical results trend in the opposite direction; there were clear inhibitory effects. The post-reciprocal region in the *Ungrammatical-Interference* condition was read at a comparable or slightly slower rate than the processing of the reciprocal in the *Ungrammatical-NoInterference* condition. Despite the fact that our study potentially lacks the power to observe an interference effect, we are more secure in our conclusion that there is a lack of facilitatory interference in light of the direction of the numerical trend toward an interaction in the post-reciprocal region.

Two words downstream from the reciprocal, reading times were longest when the local subject did not match the features of the reciprocal but the features of the distractor did match the reciprocal's number features.^2^ We discuss this effect below because although it is inconsistent with the predictions of our simulations, it does potentially indicate that the distractor's morphological features may affect overall processing of the reciprocal.

The mechanism by which the distractor exerts inhibitory influence on reciprocal licensing is unclear. It is commonly assumed that inhibitory interference should occur when multiple items in memory match a retrieval cue (e.g., Badecker and Straub, 2002; Lewis and Vasishth, 2005; Van Dyke and McElree, 2011). Yet, we observed inhibition in the absence of a multiple-match configuration: the main subject matched the positional cue and the distractor matched the number cue. This suggests that the mechanism used to explain inhibition in multiple-match cases (e.g., the fan effect in Lewis and Vasishth's, 2005 model), is not the appropriate explanation for our finding. We consider three possible explanations of this inhibitory effect and the role that number features play in guiding initial retrieval under each scenario.

The first possible interpretation of the inhibitory effect links the slightly delayed slowdown to erroneous retrieval of the distractor during initial memory access. The increased reading times in our SPR experiment might reflect initial misretrieval of the distractor based on its morphological overlap with the probe, followed by the increased processing cost of inhibiting that distractor. This line of reasoning has been pursued by Patil et al. (2012) and Chen et al. (2012) to explain inhibitory effects in reflexive licensing. We consider this interpretation unlikely for the present data because we see no evidence of the erroneous retrieval on which the explanation is predicated. In light of subject-verb agreement and NPI licensing effects, we would expect initial misretrieval to result in some degree of facilitation, however fleeting, that would be observable in the self-paced reading times. These facilitatory interference effects consistently yield large effects on reading times in studies of other linguistic dependencies. No such facilitation was observed prior to the point of inhibitory interference in the current study.

The inhibitory effect might also be explained in terms of *cue-confusability*, as defined by Jäger et al. (2014). The proposal rests on the speculation that cues that reliably co-occur in specific retrieval contexts can be confused (less effectively deployed). In reciprocal licensing the clause and plural cues are reliably associated because both cues should be selected whenever a reciprocal is encountered. This contrasts with cue association in reflexive licensing where specific gender cues (e.g., masculine and feminine) and the clause-mate cue co-occur less reliably, e.g., it is not the case that reflexive licensing uniformly uses masculine gender. According to the proposal, confusion is more likely to occur in reciprocal licensing than in reflexive licensing. Although we note that this is a possibility in principle, we believe that the notion of cue-confusability or the mechanism by which confusion creates retrieval interference has not been sufficiently articulated to be thoroughly evaluated.

The third alternative interpretation of the effect connects the slowdown to the influence of an abandoned early garden-path parse that analyzed the distractor as a constituent of the main clause. The previous partial parse could provide an appropriately marked antecedent for the reciprocal, but would fail to provide a coherent global parse. There are no grammatical re-parses of the sentence that would allow the distractor to be reanalyzed as an appropriate antecedent for the reciprocal. We hypothesize that resolving the tension between attempting to license the reciprocal and building a globally grammatical parse of the sentence is the source of the observed interaction. The misparse is expected to intrude on the processing of the reciprocal in the *Ungrammatical-Interference* condition, where consideration of the reparse would result in a structurally appropriate, feature-matching antecedent for the reciprocal.

We favor the interpretation that this inhibitory effect reflects the influence of the mis-parse on repair strategies that are triggered by failure of initial antecedent retrieval (as proposed for similar effects by, Sturt, 2003; Chow et al., 2014). On this interpretation the failure to retrieve an appropriate antecedent for the reciprocal would initiate a more liberal search for a feature-matching phrase, or would attempt to find an alternative parse for the sentence under which the reciprocal could be grammatically bound. These repair procedures are argued to be less constrained by the structure of the previous parse (and therefore structural constraints), perhaps reflecting uncertainty in the structural analysis in light of the error signal. This scenario attributes the increase in reading times to interference, but not interference that occurs during antecedent retrieval. Rather, the locus of interference lies in retrievals associated with syntactic revision and reanalysis. It is also possible that the distractor in the mis-parsed sentence could contribute interference at retrieval time, a possibility that would be consistent with the numerical trend toward an interaction in the post-reciprocal region. We acknowledge that the present study cannot distinguish between these two options.

In sum, our SPR experiment failed to find the characteristic profile of facilitatory interference that has been found in other studies on the construction of subject-verb agreement, NPI-licensing, and control dependencies and is predicted under a cue-based retrieval model that uses morphological cues to access potential antecedents for a local anaphor. Instead, a feature-matching distractor triggered a delayed inhibitory effect when the local subject could not antecede the reciprocal in Hindi. We argued that this process was not an indication of interference during antecedent retrieval, but rather interference during a repair process subsequent to antecedent retrieval.

## General discussion

The purpose of the present study was to assess whether syntactic cues are given priority over morphological cues in the retrieval of antecedents of pre-verbal reciprocals in Hindi. Investigating the processing of Hindi reciprocals helps to establish whether the absence of facilitatory interference effects from morphologically-matched distractors in previous experiments was due to a confound of anaphor position. We hypothesized that if the absence of interference were solely due to the post-verbal position of the anaphor, and not prioritization of syntactic cues, interference would be observable in the retrieval of an antecedent for a pre-verbal anaphor in Hindi.

In our self-paced reading study native Hindi speaking participants resolved a local reciprocal dependency more quickly when the main clause subject was plural than when no grammatical antecedent was present. The presence of a feature-matching distractor did not induce reliable effects of facilitatory interference when the local subject did not match the reciprocal in features. These findings are consistent with a general lack of facilitation in the licensing of local anaphors found in previous work (e.g., Sturt, 2003; Xiang et al., 2009; Dillon et al., 2013), and with lack of interference during local anaphor licensing more generally (e.g., Nicol and Swinney, 1989; Clackson, 2011). The presence of a feature-matching distractor produced a delayed inhibitory effect when an appropriate antecedent for the reciprocal could not be found. We reasoned that the inhibitory effect in our experiment might have arisen as a result of error-driven repair strategies, and not from participants accessing the distractor during initial antecedent retrieval.

The empirical results of our SPR experiment were compared against the results of a series of simulations that modeled latencies and error rates of a cue-based retrieval process that used equally-weighted morphological and positional cues to retrieve antecedents of a local anaphor. The empirical results did not align with the simulations' prediction that there should be facilitatory interference between ungrammatical conditions.

Overall, the results lend support to the hypothesis that the lack of facilitatory interference in local anaphor antecedent retrieval is not primarily determined by an anaphor's post-verbal position. In particular, the Hindi results appear to be incompatible with a number of the possible ways in which verbal adjacency could influence retrieval of antecedents for local anaphors discussed. The results cast doubt on explanations that rely on recent reactivation of the grammatical antecedent immediately before the reciprocal. In the Hindi materials there is no point at which retrieval of the subject is required between the distractor and when the reciprocal is encountered.

The results are consistent with models of cue-based antecedent retrieval that prioritize syntactic information in one manner or another. As noted in the introduction, a parser could be said to prioritize syntactic cues by assigning them greater weight than morphological cues, or by using syntactic cues exclusively.

Because some dependencies display facilitatory interference effects while others do not, it would appear that retrieval does not consistently prioritize positional cues. One question that arises is how the parser determines when it should prioritize syntactic cues. Rational models often assume that retrieval uses a set of cues and weights that maximizes the probability of retrieving the target, while minimizing the chances of interference. It is important to note that the optimal cue set for meeting both of these goals may change as a function of (i) the dependency being computed and (ii) the local syntactic context. Therefore, strategic considerations that take the local context into account may comprise an important part of the cue selection procedure. We term different solutions that the parser could adopt *retrieval strategies*.

The parser could adopt one of two strategies that make different use of morphological cues during local antecedent retrieval. First, the parser could uniformly prioritize syntactic cues for all instances of local antecedent retrieval regardless of syntactic context. Dillon et al. (2013) proposed that the parser implements such a retrieval strategy. According to these authors, local antecedent retrieval only uses structural cues.

An alternative to this proposal is that the parser could condition the use of morphological cues on the local syntactic context of the anaphor that triggers retrieval, as proposed by Kush (2013). The intuition behind this proposal stems from the observation that in certain environments structural cues alone may not suffice to identify a unique antecedent for a local anaphor. If the subject of the local clause is the anaphor's only co-argument, as it is in (15), then syntactic cues are sufficient to guarantee its retrieval. However, if there exists an additional co-argument that precedes the anaphor as in (16), a syntactic cue like the clause feature would not be able to distinguish the appropriate antecedent (*the boys*) from the structurally appropriate, but feature-mismatching NP *Mary*.

(15) *The boys* spoke with *each other*.
(16) Mary introduced *the boys* to *each other*

Kush (2013) proposed that a parser that could determine the number of clause-mates that preceded a local anaphor might use morphological cues to help guarantee retrieval of an appropriate antecedent. Determining whether the local subject is the anaphor's only clause-mate should be possible by consulting the local syntactic context. When processing English reflexives in direct object position, the anaphor's adjacency to the verb would be sufficient. In Hindi, verbal adjacency cannot be exploited to make such a determination. Kush (2013) proposed that the decision could be made if cue selection had access to the phrase structure rule being used to incrementally parse the input sentence. In cases where the anaphor is the first NP encountered during the incremental parse of the VP, the phrase structure predicted for the VP should not contain co-argument NPs. On the other hand, if the parser encounters a non-subject co-argument that precedes the reciprocal, the PS rule for the VP would reflect its presence and cue selection could determine that the clause index cue would no longer provide diagnostic access to the local subject.

If the parser adopts this retrieval strategy interference effects are predicted to emerge when there are non-subject clause-mates that precede a local anaphor. This proposal is consistent with recent findings from Wagers and colleagues, which suggest that that resistance to interference is, in fact, selectively conditioned on whether the anaphor is encountered after another co-argument (King et al., 2012). Under this interpretation, interference should emerge if a co-argument preceded the reciprocal in Hindi, as in (17). We leave testing this prediction to future work.

(17) ^*^Larke-ne Mary-ko baccoN-ki party me ek-dusre ke-bare-me bataayaa.
Boy-Erg Mary-Acc kids' party in one-another about told.
^*^The boy told Mary during the kids' party about *each other*.

## Conclusion

In this paper we asked whether the absence of intrusive licensing during local anaphor antecedent retrieval is restricted to post-verbal anaphors, or whether the lack of interference indicates a more general cross-linguistic state of affairs. We investigated the effect of a feature-matching distractor on the processing of unlicensed pre-verbal reciprocals in Hindi and found no indication of facilitatory interference. The results suggest that antecedent retrieval's ability to accesses the syntactically appropriate subject when licensing a local anaphor does not depend on direct verbal adjacency between the anaphor and its verb. The results appear to be better explained by a cue-based retrieval process that prioritizes, or exclusively uses, structural cues over morphological features. Finally, although we did not find evidence that a feature-matching distractor facilitates the processing of an unlicensed reciprocal, it did appear that a distractor might exert an inhibitory influence on some stage of reciprocal resolution. Future work should test whether this inhibition is a general effect, or whether its appearance is related to properties of the materials used here.

### Conflict of interest statement

The authors declare that the research was conducted in the absence of any commercial or financial relationships that could be construed as a potential conflict of interest.

## Footnotes

^1^Total source activation took one of three values across our simulations: 1.0, 1.25, 1.5. Four values were possible for the activation noise parameter: 0.3, 0.4, 0.5, 0.6. Three values were used for the fan parameter: 1.0, 1.5, 2.0, centered at the default value of 1.5 (Lewis and Vasishth, 2005). Three decay rates were used: 0.5 (the default rate of decay; Lewis and Vasishth, 2005), 0.25, and.001. Finally, we used three values for the match-penalty parameter: −0.2, −0.4, and −0.6.

^2^We expect that this effect would be stronger with a larger sample.

## References

[B1] Anderson J. R. (1989). A rational analysis of human memory, in Varieties of Memory and Consciousness: Essays in Honor of Endel Tulving, eds Roediger H. III Craik F. (Hillsdale, NJ: Erlbaum), 195–210.

[B2] Anderson J. R. (1990). The Adaptive Character of Thought. Hillsdale, NJ: Erlbaum.

[B3] Anderson J. R. Milson R. (1989). Human memory: an adaptive perspective. Psychol. Rev. 96, 703–719 10.1037/0033-295X.96.4.703

[B4] Anderson J. R. Schooler L. (1991). Reflections of the environment in memory. Psychol. Sci. 2, 396–408 10.1111/j.1467-9280.1991.tb00174.x

[B5] Baayen R. H. Davidson D. J. Bates D. M. (2008). Mixed-effects modeling with crossed random effects for subjects and items. J. Mem. Lang. 59, 390–412 10.1016/j.jml.2007.12.005

[B6] Badecker B. Lewis R. (2007). A new theory and computational model of working memory in sentence production: agreement errors as failures of cue-based retrieval, in Paper Presented at the 20th Annual CUNY Sentence Processing Conference (San Diego; La Jolla, CA: University of California).

[B7] Badecker W. Straub K. (2002). The processing role of structural constraints on the interpretation of pronouns and anaphors. J.Exp. Psychol. Learn. Mem. Cogn. 28, 748–769. 10.1037/0278-7393.28.4.748 12109766

[B8] Barr D. J. Levy R. Scheepers C. Tily H. J. (2013). Random effects structure for confirmatory hypothesis testing: keep it maximal. J. Mem. Lang. 68, 255–278. 10.1016/j.jml.2012.11.001 24403724 PMC3881361

[B9] Bhatt R. Dayal V. (2007). Rightward scrambling as rightward remnant movement. Linguist. Inq. 38, 287–301 10.1162/ling.2007.38.2.287

[B10] Chen Z. Jäger L. Vasishth S. (2012). How structure-sensitive is the parser? Evidence from Mandarin Chinese, in Empirical Approaches to Linguistic Theory: Studies of Meaning and Structure, eds Stolterfoht B. Featherston S. (Berlin: Studies in Generative Grammar, Mouton de Gruyter), 43–62.

[B11] Chomsky N. (1981). Lectures on Government and Binding. Berlin: Mouton de Gruyter.

[B48] Chow W.-Y. Lewis S. Phillips C. (2014). Immediate sensitivity to structural constraints in pronoun resolution. Front. Psychol. 5:630. 10.3389/fpsyg.2014.00630 25018739 PMC4073625

[B12] Clackson K. (2011). Reflexives and Pronouns in Sentence Processing: an Experimental Study of Children and Adults. Colchester, UK: Doctoral Dissertation, University of Essex.

[B13] Dayal V. (1994). Binding facts in Hindi and the scrambling phenomenon, in Theoretical Perspectives on Word Order Issues in South Asian Languages, eds Butt M. King T. H. Ramchand G. (Stanford, CA: CSLI Publications), 237–262.

[B14] Dillon B. Mishler A. Slogett S. Phillips C. (2013). Contrasting intrusion profiles for agreement and anaphora: experimental and modeling evidence. J. Mem. Lang. 69, 85–103 10.1016/j.jml.2013.04.003

[B15] Drenhaus H. Frisch S. Saddy D. (2005). Processing negative polarity items: when negation comes through the backdoor, in Linguistic Evidence: Empirical, Theoretical, and Computational Perspectives, eds Kepser S. Reis M. (Berlin: Mouton de Gruyter), 145–165.

[B17] Inoue A. (1991). A Comparative Study of Parsing in English and Japanese. PhD dissertation. Mansfield, CT: University of Connecticut.

[B18] Jäger L. Engelmann F. Vasishth S. (2014). Inhibitory interference in reflexives: Evidence for cue confusability, in Poster AMLaP 2014 (Edinburgh).

[B19] Just M. A. Carpenter P. A. Woolley J. D. (1982). Paradigms and processes and in reading comprehension. J. Exp. Psychol. Gen. 3, 228–238. 10.1037/0096-3445.111.2.228 6213735

[B20] King J. Andrews C. Wagers M. (2012). Do reflexives always find grammatical antecedents for themselves? in Poster, 25th Annual CUNY Human Sentence Processing Conference (New York, NY).

[B21] Kush D. (2013). Respecting Relations: Memory Access and Antecedent Retrieval in Incremental Sentence Processing. Doctoral Dissertation, University of Maryland, College Park.

[B22] Lasnik H. (2002). Clause-mate conditions revisited. Glot Int. 6, 94–96.

[B23] Lewis R. Vasishth S. (2005). An activation-based model of sentence processing as skilled memory retrieval. Cogn. Sci. 29, 375–419. 10.1207/s15516709cog0000_25 21702779

[B24] Lewis R. Vasishth S. Van Dyke J. (2006). Computational principles of working memory in sentence comprehension. Trends Cogn. Sci. 10, 447–454. 10.1016/j.tics.2006.08.007 16949330 PMC2239011

[B25] Martin A. E. McElree B. (2008). A content-addressable pointer mechanism underlies comprehension of verb-phrase ellipsis. J. Mem. Lang. 58, 879–906. 10.1016/j.jml.2007.06.01019686017

[B26] Martin A. E. McElree B. (2009). Memory operations that support language comprehension: evidence from verb-phrase ellipsis. J. Exp. Psychol. Learn. Mem. Cogn. 35, 1231–1239. 10.1037/a0016271 19686017 PMC2849635

[B27] Mazuka R. Itoh K. (1995). Can Japanese speakers be led down the garden path? in Japanese Sentence Processing, eds Mazuka R. Nagai N. (Hillsdale, NJ: Lawrence Erlbaum Associates), 295–329.

[B28] McElree B. (2000). Sentence comprehension is mediated by content-addressable memory structures. J. Psycholinguist. Res. 29, 111–123. 10.1023/A:1005184709695 10709178

[B31] McElree B. Foraker S. Dyer L. (2003). Memory structures that subserve sentence comprehension. J. Mem. Lang. 48, 67–91. 10.1016/S0749-596X(02)00515-621702779

[B32] Miyamoto E. T. (2003). Reanalysis of clause boundaries in Japanese as a constraint-driven process. Lang. Speech 46, 23–50. 10.1177/00238309030460010301 14529110

[B33] Mohanan T. (1994). Argument Structure in Hindi. Stanford, CA: CSLI Publications.

[B34] Nairne J. S. (1990). A feature model of immediate memory. Mem. Cognit. 18, 251–269. 10.3758/BF03213879 2192233

[B36] Nicol J. Swinney D. (1989). The role of structure in coreference assignment during sentence comprehension. J. Psycholinguist. Res. 18, 5–19. 10.1007/BF01069043 2647962

[B37] Parker D. Lago S. Phillips C. (2012). Retrieval interference in the resolution of Anaphoric PRO, in Talk, 35th GLOW Conference (Potsdam).

[B38] Patil U. Vasishth S. Lewis R. (2012). Retrieval interference in syntactic processing: The case of reflexive binding in English. Manuscript, Potsdam: University of Potsdam. 10.3389/fpsyg.2016.00329 PMC4881398 27303315

[B39] Pearlmutter N. J. Garnsey S. M. Bock K. (1999). Agreement processes in sentence comprehension. J. Mem. Lang. 41, 427–456 10.1006/jmla.1999.2653

[B40] Reinhart T. (1983). Anaphora and Semantic Interpretation. London: Croom Helm.

[B41] Sturt P. (2003). The time-course of the application of binding constraints in reference resolution. J. Mem. Lang. 48, 542–562 10.1016/S0749-596X(02)00536-3

[B42] Van Dyke J. A. (2007). Interference effects from grammatically unavailable constituents during sentence processing. J. Exp. Psychol. Learn. Mem. Cogn. 33, 407–430. 10.1037/0278-7393.33.2.407 17352621 PMC2077343

[B43] Van Dyke J. A. McElree B. (2006). Retrieval interference in sentence processing. J. Mem. Lang. 55, 157–166. 10.1016/j.jml.2006.03.007 18209744 PMC2206541

[B44] Van Dyke J. A. McElree B. (2011). Cue-dependent interference in comprehension. J. Mem. Lang. 65, 247–263. 10.1016/j.jml.2011.05.002 21927535 PMC3171743

[B45] Vasishth S. Brssow S. Lewis R. Drenhaus H. (2008). Processing Polarity: how the ungrammatical intrudes on the grammatical. Cogn. Sci. 32, 685–712. 10.1080/03640210802066865 21635350

[B46] Wagers M. Lau E. F. Phillips C. (2009). Agreement attraction in comprehension: representations and processes. J. Mem. Lang. 61, 206–237. 10.1016/j.jml.2009.04.00225258471

[B47] Xiang M. Dillon B. Phillips C. (2009). Illusory licensing effects across dependency types: ERP evidence. Brain Lang. 108, 40–55. 10.1016/j.bandl.2008.10.002 19007980

